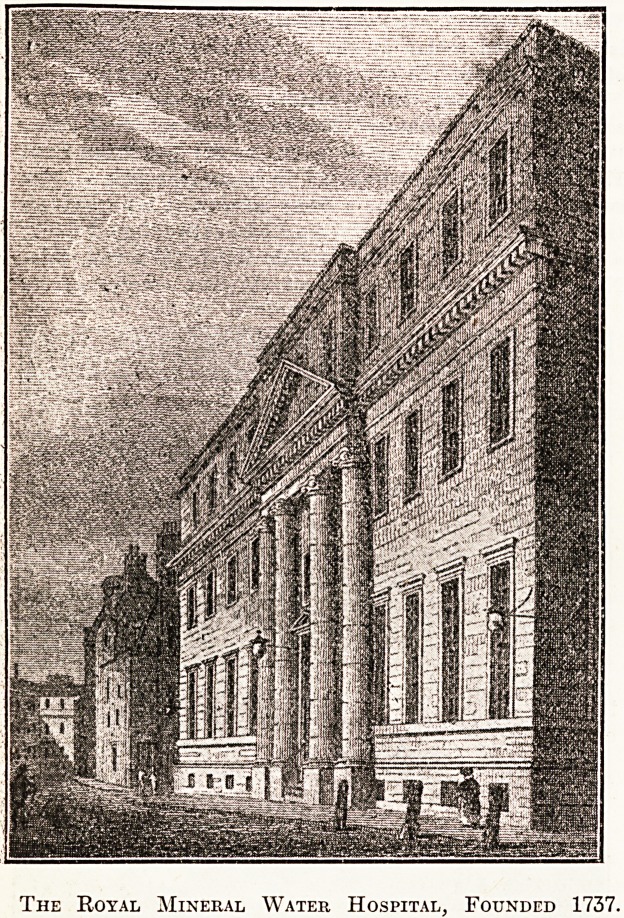# Sidelights on the Mineral Water Hospital, Bath

**Published:** 1914-03-07

**Authors:** 


### BEAU NASH AS A HOSPITAL OFFICER.

### Sidelights on the Mineral Water Hospital, Bath.

THE privilege of "taking the waters" of Bath has never by any means been confined to those of wealth and position; for as early as the reign of Queen Elizabeth

THE INVENTOR OF "BATH OLIVERS."

an Act of Parliament gave permission to the poor to obtain the free use of its healing springs, and it was not until the year 1714 that the Act was repealed. The city had by

RICHARD (BEAU) NASH, THE M.C. OF BATH.

this time become the Mecca of vagrants, who, though they came ostensibly for the "cure," had ulterior motives. It was decided that steps should be taken to ensure the treatment of those who were really worthy, and with this object in view a project was launched to found a hospital. The promoters were unable at first to agree, with the result that it was not until 1737 that the foundation-stone of the General Hospital was laid by the Right Hon. William Pulteney, afterwards Earl of Bath, whose memory is perpetuated to-day by the names of various roads and streets adjoining the city.

Foremost among the supporters and promoters of the scheme was the celebrated Master of Ceremonies of Bath, "Beau" Nash, who acted as one of the treasurers from 1742-1761; he is also reputed to have collected a large sum of money, and taken a lively interest in the construction of the building. A statue of him exists there holding a plan of the hospital in his right hand. Others besides Nash entered into the spirit of the new enterprise, for

RALPH ALLEN, WHO DISCOVERED THE BATH QUARRIES.

among its supporters are to be found many names famous in the history of Bath. Ralph Allen, who discovered the value of the local stone and opened large quarries, gave all the stone used in the original building, as well as a generous donation of money. Sir Richard Steele, Dr. Oliver, the famous originator of the Bath Oliver biscuit, and John Wood, the architect, deserve mention also. The latter drew all the plans and specifications for the hospital free of cost, and in 1738, by an Act of Parliament, the hospital was founded and ninety persons were named as trustees; the Act further directed that there should always be a President, three treasurers, and a committee of thirty-two, to be elected annually on May 1. The committee was to meet weekly, and it was on the eve of Christmas, 1741, that the first matron was appointed, and the hospital was opened in the following year. In 1861, on account of pressure of space and extent of work, additional premises were built; during these operations some Roman remains in the shape of a piece of pavement were discovered; they are now to be seen within the walls of the hospital.

The present building has accommodation for 150 persons, eighty-three males and sixty-seven females; and it is worthy of note that no letter, nomination, recommendation, or influence of any description is required to gain admission; it is required merely to show that the person is not in a position to incur the expense of the mineral water treatment, and that there is a reasonable possibility of beneficial results, to secure treatment at the hospital, the average stay at which is six weeks.

The Royal Mineral Water Hospital, Founded 1737.

## Figures and Tables

**Figure f1:**
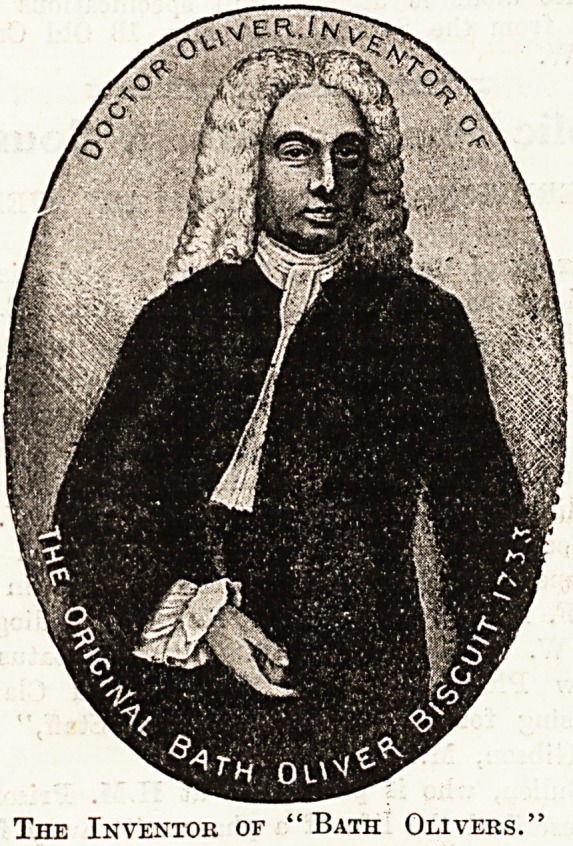


**Figure f2:**
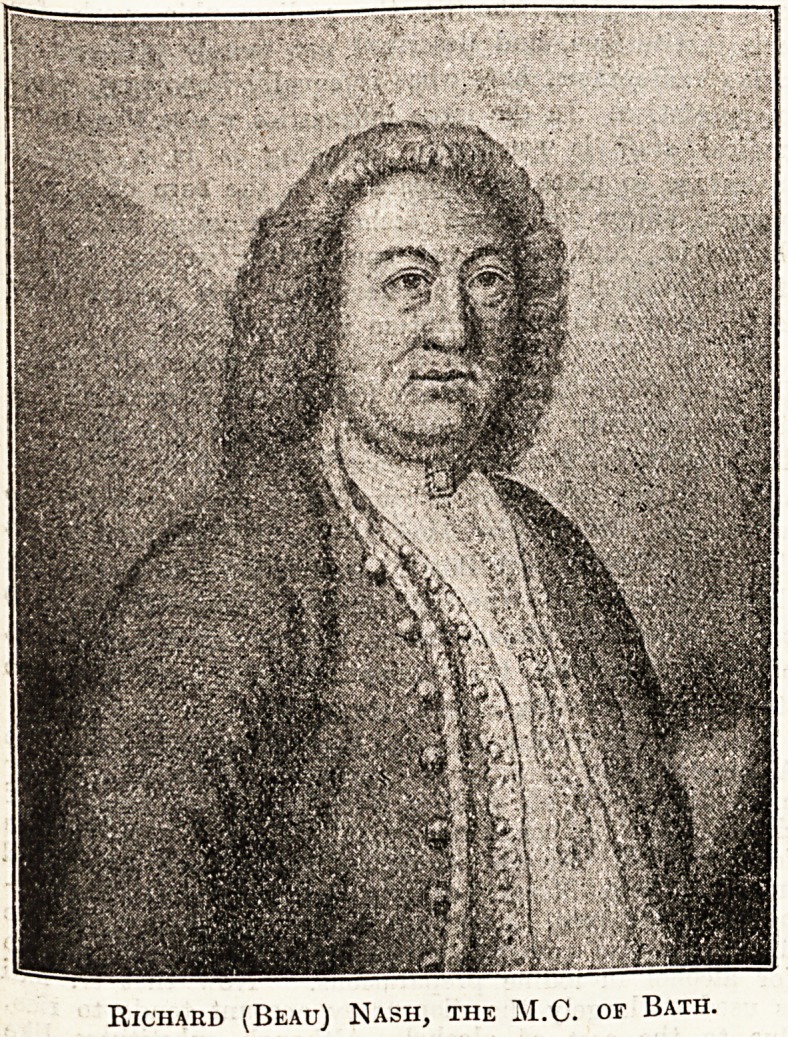


**Figure f3:**
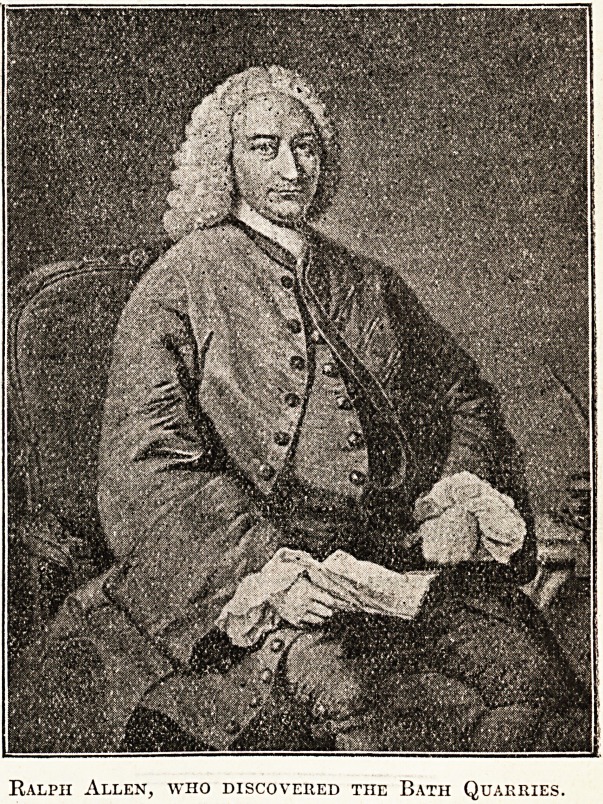


**Figure f4:**